# A cross sectional survey on social, cultural and economic determinants of obesity in a low middle income setting

**DOI:** 10.1186/s12939-015-0140-8

**Published:** 2015-01-17

**Authors:** Ambepitiyawaduge Pubudu De Silva, Sudirikku Hennadige Padmal De Silva, Rashan Haniffa, Isurujith Kongala Liyanage, Kosala Saroj Amarasiri Jayasinghe, Prasad Katulanda, Chandrika Neelakanthi Wijeratne, Sumedha Wijeratne, Lalini Chandika Rajapakse

**Affiliations:** Department of Community Medicine, Faculty of Medicine, University of Colombo, Colombo, Sri Lanka; Centre for Tropical Medicine, University of Oxford, Oxford, UK; Department of Para Clinical Sciences, Faculty of Medicine, General Sir John Kotelawala University, Ratmalana, Sri Lanka; Department of Clinical Medicine, Faculty of Medicine, University of Colombo, Colombo, Sri Lanka; Department of Obstetrics & Gynaecology, Faculty of Medicine, University of Colombo, Colombo, Sri Lanka

**Keywords:** Prevalence of obesity, Socioeconomic and cultural determinants of obesity, Obesity in plantation sector, Obesity in Muslims

## Abstract

**Introduction:**

Obesity is an increasing problem in South Asian countries and Sri Lanka is no exception. The socioeconomic determinants of obesity in Sri Lanka, and in neighbouring countries are inadequately described. Aim was to describe social, cultural and economic determinants of obesity in a representative sample from Kalutara District in Sri Lanka.

**Methods:**

This was a cross sectional descriptive study conducted among adults aged 35–64 years. A representative sample was selected using stratified random cluster sampling method from urban, rural and plantation sectors of Kalutara District. Data were collected using a pre-tested questionnaire. A body mass index of 23.01 kg/m^2^-27.50 kg/m^2^ was considered as overweight and ≥27.51 kg/m^2^ as obese. Waist circumference (WC) of ≥ 90 cm and ≥80 cm was regarded as high for men and women respectively. Significance of prevalence of obesity categories across different socio-economic strata was determined by chi square test for trend.

**Results:**

Of 1234 adults who were screened, age and sex adjusted prevalence of overweight, obesity and abdominal obesity (high WC) were 33.2% (male 27.3%/female 38.7%), 14.3% (male 9.2%/female 19.2%) and 33.6% (male 17.7%/female 49.0%) respectively. The Muslims had the highest prevalence of all three obesity categories. Sector, education, social status quintiles and area level deprivation categories show a non linear social gradient while income shows a linear social gradient in all obesity categories, mean BMI and mean WC. The differences observed for mean BMI and mean WC between the lowest and highest socioeconomic groups were statistically significant.

**Conclusion:**

There is a social gradient in all three obesity categories with higher prevalence observed in the more educated, urban, high income and high social status segments of society. The higher socioeconomic groups are still at a higher risk of all types of obesity despite other public health indicators such as maternal and infant mortality displaying an established social gradient.

**Electronic supplementary material:**

The online version of this article (doi:10.1186/s12939-015-0140-8) contains supplementary material, which is available to authorized users.

## Introduction

The obesity epidemic is a worldwide public health problem [1,2]. It is a major risk factor for non-communicable diseases (NCDs) causing diabetes, cardiovascular disease, cancer and premature death [1,3]. In higher income countries (HICs) the overall prevalence of obesity among men and women were 18.2 – 19.9 and 21.2 - 23.2 respectively while in India, China and Sub Saharan African regions this varies from 1.8 to 3.1 and 3.9 to 10.7 respectively [4]. Although a high prevalence of obesity is observed in HICs a larger burden rests in lower income countries (LICs)/lower middle income countries (LMICs) as most of the obese (and non-obese) population resides in these countries [3-5]. Further these LICs/LMICs demonstrate a continual rise in obesity prevalence [3,6].

The publications on obesity in other countries in the South Asian regions have not described in detail the correlations with social gradient. These were limited to associations of individual economic status and education with obesity [7-12]. The social gradients in obesity in HICs, give rise to inequalities in health outcomes and worsen the health of disadvantaged groups [13,14]. In order to tackle the burden of obesity it is important to understand the social determinants of obesity. Knowledge of obesity prevalence within social groups will provide insights into causal pathways and avenues for possible public health intervention. Any social gradient of obesity prevalence or its change from established patterns is important in any interventions directed towards minimising these conditions, especially as groups at the bottom of the social gradient already have established disadvantages in other spheres of well-being such as income, wealth or education [13]. Using a social determinants approach to obesity would provide an opportunity for sustainable and equitable outcomes [14].

We have published the findings of social determinants of diabetes mellitus in a representative sample from Kalutarta District, Sri Lanka [15]. We now describe in the same sample using additional data collected, the prevalence of obesity, and its socioeconomic and cultural determinants.

## Methods

The detailed methodology of this cross sectional survey has already been described [15]. The study was conducted in Kalutara district (a district with urban, rural and estate population in Sri Lanka) among 35 to 64 year olds. A sample of 1300 was shown to be adequate to detect an overweight and central obesity prevalence of 25% [7] with a margin of error at 3.5% and α error at 5% with consideration of 10% dropout rate and a cluster effect of 2. A random, stratified cluster sampling method was used to select the participants. The first level of stratification was at urban, rural and estate sectors with rural and estate sectors being over sampled. The primary sampling unit was the Grama Niladari Division (GND), which is the lowest village level administrative division in Sri Lanka. The selection of GNDs within the sector was probability proportionate to the size of the 35 to 64 years population. Within the GNDs 20 households were randomly selected using the electoral registry and an eligible individual was selected randomly from each household. Pregnant and lactating females, institutionalized individuals and those on prolonged treatment with drugs known to cause diabetes mellitus were excluded.

The body weight was measured to the nearest 100 g with Virtual and Measurements Control model VW 320, electronic digital weighing scales. The body height was measured to the nearest 0.5 cm using “Seca microtoise” steel tapes. When measuring body height, the subjects were made to stand looking straight ahead with their head, back and feet touching the vertical support [16]. The measurements were taken from the ground to the uppermost position of the head while the person was in full inspiration. The waist circumference (WC) was measured to the nearest 0.2 cm using plastic flexible (non-elastic) measuring tapes. It was measured at the midway between the lower margin of the lowest rib and the upper margin of the iliac crest, at the end of a normal expiration [17].

All measurements were taken while the patient was in light indoor clothing, without footwear or items in pockets. All measuring instruments were calibrated (zero and span calibration) before use.

Anthropometric measurements were taken by five trained Public Health Nursing Sisters (PHNSs). The training was conducted at Medical Research Institute (MRI), Colombo. All five PHNSs were standardized for measuring weight, height and waist circumference. Using a block design the variation between instruments and observers were assessed simultaneously. A block consisted of five adults in the age group of 35 to 64 years. Each PHNS took measurement of weight, height and waist circumference in all five individuals using five different sets of measuring instruments. The mean scores of measurements of the five individuals were compared with the five PHNS and the five instruments.

Inter–observer reliability of anthropometric measurements was assessed by the Pearson’s correlation coefficient (r). This was calculated for each of the anthropometric measures using the measurements made by the principal investigator and each PHNS.

The body mass index (BMI) was used as the indicator of general obesity while WC was used as the indicator of abdominal obesity. A BMI of ≤ 18.49 kg/m^2^ was regarded as underweight, 18.50 kg/m^2^ to 23.00 kg/m^2^ as desirable, 23.01 kg/m^2^ to 27.50 kg/m^2^ as overweight and ≥ 27.51 kg/m^2^ as obese [18]. WC of ≥ 90 cm and ≥80 cm was regarded as high for men and women respectively [19,20].

Measures were taken to improve the quality and accuracy of data including training and standardization of data collectors, strict adherence to operations manuals and calibration of instruments [21].

The social status index developed by De Silva [21] and the Unsatisfactory Basic Needs Index (UBNI), an area level deprivation index for Sri Lanka, developed by Satharasinghe [22] were used. All results were weighted and adjusted for age and sex of the Sri Lankan population. Significance of prevalence across different socio-economic strata was determined by chi square test for trend.

Ethical approval was obtained from the Ethics review committee of the Faculty of Medicine, University of Colombo.

## Results

Among the 1300 selected, 1234 (94.92%) participated in the study. There were no significant differences between the observers (p = 0.99) and between the instruments (p = 0.99) (Additional file 1). The levels of agreements for weight, height and WC among the five PHNS were more than 0.78 for all the measurements representing good reliability (Additional file 2).

Overall age and sex adjusted prevalence of overweight, obesity and abdominal obesity were 33.2% (391), 14.3% (167) and 33.6% (407) respectively. Table 1 describes the age and sex distribution of obesity categories, mean BMI and mean WC while the socioeconomic determinants of these are described in Table 2.

**Table 1:** **Age and sex distribution of obesity categories, BMI and WC among 35 to 64 year olds in Kalutara district**

**Age categories**	**Obesity categories**						**BMI (kgm** ^**−2**^ **)**		**WC (cm)**	
	**Overweight**		**Obese**		**Abdominal obesity**		**Mean**	**95% CI**	**Mean**	**95% CI**
	**Number**	**Percent**	**Number**	**Percent**	**Number**	**Percent**				
**Both genders**										
35 to 39 Years (n = 186)	53	30.2%	32	39.4%	62	33.0%	22.4	21.59–23.30	78.7	76.28–81.07
40 to 44 Years (n = 220)	74	31.5%	41	15.4%	87	40.3%	23.8	22.95–24.67	81.1	79.07–83.21
45 to 49 Years (n = 213)	86	44.0%	25	13.6%	80	37.5%	23.6	22.79–24.33	81.3	79.52–83.14
50 to 54 Years (n = 201)	55	31.3%	27	15.1%	60	31.1%	22.9	21.98–23.81	79.8	77.68–81.85
55 to 59 Years (n = 224)	64	29.0%	30	12.6%	71	28.0%	22.7	21.87–23.55	81.1	78.90–83.29
60 to 64 Years (n = 182)	59	32.4%	12	4.9%	47	27.3%	21.6	20.92–22.34	78.0	75.95–79.95
Total (n = 1226)	391	33.2%	167	14.3%	407	33.6%	22.9	22.70–23.18	80.1	79.47–80.68
**Male**										
35 to 39 Years (n = 107)	31	28.6%	12	7.5%	24	17.2%	21.5	20.78–22.29	79.9	77.70–82.10
40 to 44 Years (n = 111)	34	22.7%	15	16.6%	26	22.4%	22.8	21.93–23.67	82.2	79.97–84.39
45 to 49 Years (n = 104)	43	34.4%	6	4.6%	27	16.4%	22.0	21.39–22.60	80.8	79.34–82.29
50 to 54 Years (n = 99)	26	33.2%	9	7.8%	24	20.2%	21.8	21.19–22.49	80.3	78.30–82.37
55 to 59 Years (n = 111)	36	22.3%	9	10.8%	24	14.6%	21.9	21.22–22.58	81.2	79.46–82.90
60 to 64 Years (n = 90)	22	19.3%	6	6.4%	12	12.8%	21.0	20.24–21.75	80.1	78.21–82.08
Total (n = 622)	192	27.4%	57	9.2%	137	17.7%	21.9	21.46–22.36	80.8	79.57–82.01
**Female**										
35 to 39 Years (n = 79)	22	31.7%	20	23.1%	38	48.0%	23.3	22.38–24.23	77.5	74.92–80.02
40 to 44 Years (n = 109)	40	39.3%	26	21.6%	61	55.9%	24.7	23.79–25.59	80.2	78.11–82.35
45 to 49 Years (n = 109)	43	53.1%	19	22.2%	53	57.8%	25.1	24.36–25.79	81.8	79.94–83.70
50 to 54 Years (n = 102)	29	29.5%	18	21.9%	36	41.5%	23.9	22.92–24.83	79.2	77.39–81.04
55 to 59 Years (n = 113)	28	37.5%	21	14.9%	47	45.6%	23.7	22.84–24.65	81.0	78.50–83.49
60 to 64 Years (n = 92)	37	43.3%	6	3.7%	35	39.3%	22.2	21.40–22.91	76.1	73.91–78.39
Total (n = 604)	199	38.7%	110	19.2%	270	49.0%	23.9	23.40–24.44	79.4	78.09–80.67

**Table 2:** **The distribution of obesity categories, BMI and WC by selected socioeconomic factors among 35 to 64 year olds in Kalutara district**

**Characteristic**	**Obesity categories**						**BMI (kgm** ^**−2**^ **)**		**WC (cm)**	
	**Overweight**		**Obese**		**Abdominal obesity**		**Mean**	**95% CI**	**Mean**	**95% CI**
	**Number**	**Percent**	**Number**	**Percent**	**Number**	**Percent**				
**Ethnicity**										
Sinhalese	292	33.3%	122	13.5%	309	32.9%	22.8	22.49–23.21	79.9	78.97–80.77
Tamil	59	12.7%	17	3.9%	51	10.6%	21.0	20.57–21.43	75.9	73.32–78.58
Muslim	39	34.4%	28	39.6%	46	61.3%	26.3	23.99–28.52	87.5	81.66–93.27
**Sector**										
Urban	146	38.0%	74	22.2%	169	46.9%	24.0	23.58–24.46	83.6	82.47–84.66
Rural	186	33.1%	76	14.2%	186	33.4%	22.8	22.48–23.19	80.1	79.17–81.06
Plantation	59	21.3%	17	5.7%	52	17.3%	20.5	20.03–20.99	73.2	71.93–74.52
**Education category**										
No schooling	12	31.5%	5	10.0%	9	16.9%	21.3	19.28–23.39	74.6	70.12–79.12
Grade 5 or below	58	23.6%	29	13.3%	62	23.7%	22.1	21.11–23.15	77.9	75.52–80.32
Grade 6 to 10	127	25.0%	48	12.9%	129	31.1%	22.4	22.82–23.00	79.4	77.84–81.00
O/L to Grade 12	112	39.7%	44	15.8%	109	37.8%	23.5	22.81–24.23	80.6	79.14–82.17
A/L and above	61	43.3%	25	13.2%	69	39.6%	23.4	22.67–24.20	83.0	80.71–85.25
**Occupation category**										
Professional	5	21.5%	1	0.6%	4	2.3%	22.1	21.57–22.70	80.1	74.76–85.38
Technical & clerical	21	45.9%	11	12.1%	24	34.3%	23.6	22.43–24.86	83.9	80.18–87.64
Vendors & sellers	48	27.7%	25	18.6%	53	33.2%	23.2	22.20–24.25	84.7	81.94–87.41
Skilled manual workers	61	23.3%	21	8.4%	44	15.0%	21.4	20.70–22.07	77.9	76.16–79.66
Unskilled manual workers	50	24.9%	13	2.2%	36	8.5%	20.9	19.97–21.76	75.6	73.29–77.89
Retired	24	39.2%	4	7.4%	13	11.0%	22.6	21.46–23.67	83.3	80.44–86.07
Unemployed	17	21.1%	1	7.2%	11	19.4%	21.8	20.01–23.67	80.2	76.22–84.16
Housewife	151	39.8%	83	19.5%	205	52.4%	24.0	23.45–24.64	79.9	78.47–81.36
**Income category (Rs. per month)**										
<10,000	118	28.8%	39	12.7%	104	29.5%	22.4	21.65–23.05	78.1	76.36–79.76
10,000 to 30,000	211	34.7%	96	13.5%	224	34.2%	23.0	22.54–23.40	80.7	79.52–81.87
>30,000	50	35.4%	27	18.0%	66	40.3%	23.7	22.83–24.66	81.6	79.34–83.84
**Social status index**										
1st quintile (richest)	89	34.2%	42	19.9%	103	43.0%	23.8	23.97–24.55	81.2	79.51–82.99
2nd quintile	91	37.2%	41	12.5%	96	33.3%	22.8	22.09–23.48	79.9	78.17–81.70
3rd quintile	81	32.3%	35	12.4%	79	29.6%	22.6	21.90–23.21	79.6	77.70–81.44
4th quintile	77	28.1%	37	12.3%	88	30.2%	22.7	21.97–23.41	79.7	77.91–81.51
5th quintile (poorest)	43	17.6%	10	4.3%	35	13.7%	20.1	19.57–20.57	72.3	70.95–73.67
**Unsatisfactory basic needs index**										
1 (poorest)	23	26.8%	3	4.9%	17	15.6%	21.4	20.08–22.71	78.0	74.91–81.14
2	38	10.5%	13	15.7%	39	31.9%	21.6	19.93–23.36	77.5	71.74–83.29
3	67	32.3%	25	12.6%	52	26.7%	22.8	22.20–23.38	79.7	78.17–81.33
4	99	34.8%	49	11.9%	120	33.9%	22.5	21.91–23.18	79.2	77.64–80.56
5 (richest)	164	36.9%	77	20.2%	179	44.9%	24.0	23.27–24.67	82.0	80.35–83.62

Obesity prevalence was significantly higher in females when compared to males in all three (overweight, obese and abdominal obesity) categories (p < 0.001).

There was a social gradient for the prevalence of overweight, obesity, and abdominal obesity in the socioeconomic categories with chi square for trends being statistically significant (p < 0.001). The mean BMI and WC show the same clear trend within sector, education, income, social status index and UBNI groups. The 95% confident intervals of BMI and WC do not overlap for highest and lowest groups in most of these socioeconomic factors.

Table 3 describes the multivariate analysis of obesity categories with selected socioeconomic factors.

**Table 3:** **Multivariate analysis of obesity categories with selected socioeconomic factors among 35 to 64 year olds in Kalutara district**

**Characteristic**	**Obesity categories**					
	**Overweight**		**Obese**		**Abdominal obesity**	
	**Odds ratio**	**95% Confidence interval**	**Odds ratio**	**95% Confidence interval**	**Odds ratio**	**95% Confidence interval**
**Age categories**						
35 to 39 Years	1		1		1	
40 to 44 Years	1.37	0.66–2.82	1.19	0.55–2.58	1.43	0.76–2.72
45 to 49 Years	2.63*	1.23–5.61	0.88	0.36–2.14	1.62	0.79–3.30
50 to 54 Years	1.55	0.71–3.40	0.92	0.38–2.24	1.15	0.53–2.49
55 to 59 Years	1.16	0.54–2.49	0.62	0.23–1.67	1.07	0.50–2.31
60 to 64 Years	1.18	0.53–2.65	0.21	0.05–0.83	0.94	0.43–2.04
**Sex**						
Male	1		1		1	
Female	1.13	0.51–2.49	3.58*	1.15–11.15	2.28	0.94–5.56
**Ethnicity**						
Sinhalese	3.63	0.88–15.04	0.45	0.02–10.29	1.95	0.32–11.73
Tamil	1		1		1	
Muslim	8.04*	1.37–47.10	1.82	0.08–43.66	6.18	0.82–46.41
**Sector**						
Urban	0.08	0.01–0.48	1.15	0.05–26.77	0.19	0.03–1.27
Rural	0.06	0.01–0.34	0.96	0.04–22.78	0.15	0.02–0.97
Plantation	1		1		1	
**Education category**						
No schooling	1		1		1	
Grade 5 or below	1.22	0.27–5.53	3.14	0.24–41.86	3.93	0.51–30.57
Grade 6 to 10	1.10	0.26–4.57	2.66	0.21–34.17	6.36	0.84–48.11
O/L to Grade 12	2.77	0.65–11.76	2.89	0.21–39.46	8.39*	1.09–64.71
A/L and above	2.59	0.56–11.90	2.29	0.16–32.34	10.82*	1.34–87.44
**Occupation category**						
Professional	0.98	0.08–12.23	0.39	0.01–11.34	0.20	0.02–1.84
Technical & clerical	1.49	0.41–5.41	7.06	0.65–76.55	4.24	0.78–23.04
Vendors & sellers	1.05	0.37–2.96	16.27*	2.22–119.46	6.67*	1.53–29.13
Skilled manual workers	0.72	0.28–1.89	7.13*	1.01–50.65	2.88	0.69–12.12
Unskilled manual workers	1		1		1	
Retired	1.22	0.33–4.52	10.92	0.91–131.04	1.46	0.24–8.70
Unemployed	0.81	0.20–3.35	1		2.06	0.28–15.31
Housewife	2.18	0.78–6.08	6.20	0.81–47.37	8.29*	2.35–29.28
**Income category (Rs. per month)**						
<10,000	1		1		1	
10,000 to 30,000	1.02	0.63–1.66	0.96	0.50–1.85	1.16	0.71–1.90
>30,000	1.23	0.56–2.69	2.36	0.88–6.31	2.38*	1.1–5.17
**Social status index**						
1st quintile (richest)	4.82*	1.12–20.76	3.57	0.61–20.78	1.95	0.51–7.45
2nd quintile	4.77*	1.17–19.41	2.40	0.44–13.02	1.76	0.48–6.48
3rd quintile	4.92*	1.22–19.90	2.32	0.44–12.57	1.84	0.52–6.52
4th quintile	3.75*	1.15–12.21	3.11	0.77–12.57	2.84	0.99–8.15
5th quintile (poorest)	1		1		1	
**Unsatisfactory basic needs index**						
1 (poorest)	1		1		1	
2	0.28	0.06–1.41	3.85	0.47–31.73	3.00	0.68–13.26
3	1.33	0.54–3.28	3.36	0.70–16.19	2.59	0.97–6.90
4	1.50	0.60–3.76	2.31	0.45–11.87	3.36*	1.20–9.39
5 (richest)	1.47	0.55–3.95	3.52	0.68–18.15	4.60*	1.60–13.25

*p < 0.05.

**Figure 1:**
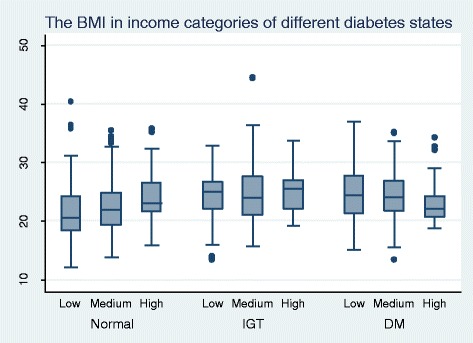
**The distribution of BMI in relation to income and diabetes status among 35 to 64 year olds in Kalutara district.**

**Figure 2:**
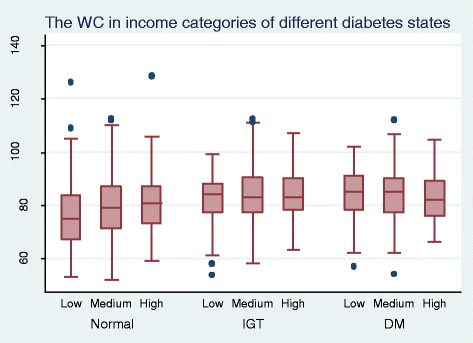
**The distribution of WC in relation to income and diabetes status among 35 to 64 year olds in Kalutara district.**

Figure 1 illustrates the distribution of BMI in relation to income and diabetes status while Figure 2 demonstrates the distribution of WC in relation income and diabetes status. The mean BMI and WC show a linear social gradient among income categories in non-diabetics.

## Discussion

Our paper demonstrates that the prevalence of obesity in Kalutara District is higher than previously reported figures for Sri Lanka, with evidence to support a rising trend, and that obesity categories show a non linear social gradient with the highest effected groups being females, urban dwellers, the high socio economic category, and the Muslim community.

The response rate of study participants was high (94.4%) and thus the participation bias is kept minimal. The study sample was comparable (age groups and gender) to the study population in Kalutara district [15]. The variations between instruments and observers for anthropometric measurements and the levels of agreements were acceptable [23].

Our prevalence for overweight, obesity and central obesity (33.2%, 14.2% and 33.6%) are higher than previously reported studies (though using slightly different criteria for obesity categorization) in Sri Lanka and the trend for increase persists [7,24-26]. The early studies in 1990 reported obesity rates of 7% for males and 13.4% for females while in 2006, the prevalence rates of overweight, obesity and central obesity were 25.2%, 9.2% and 26.2% [7,24,]. Some of these studies were limited to small sample sizes in a few regions [24,27]. Larger studies conducted in 2005/6 showed a high burden of obesity, with especially high proportions observed among females of all age categories and in the urban sector [7,25]. The female preponderance of obesity in Sri Lanka may contribute to the high prevalence of non-communicable diseases amongst women [28].

The prevalence of obesity shows wide variation in the South Asian region. The reported figures for Bangladesh were lesser than Sri Lankan figures [11]. Pakistani rates for overweight and obesity (28.2% for females 22% for males) are similar to our findings [12]. In South India the prevalence of obesity and abdominal obesity ranged from 35.1% to 56.2% among males and females which were higher than our figures [29]. Prevalence rates of overweight and obesity reported in Nepal (59.1% for males and 61.8% for females) and Maldives (60.8% of males and 65.5% of females) were much higher than our findings [8,10].

With regard to socio economic status and obesity Katulanda demonstrated increasing odds ratios for obesity with increasing income levels similar to our findings [7]. Similar findings were observed for the prevalence of diabetes mellitus and impaired glucose tolerance in Sri Lanka in 1993 and in 2012 even as Sri Lanka moved from a LIC to a LMIC [15,27,30].

Higher prevalence of obesity was seen in low socio economic strata for HICs while the reverse was observed in LICs and LMICs [31]. Obesity occurring in developing countries was shown to affect the affluent [3]. South Asian settings show mixed results. Pakistan being a LMIC demonstrates a gradient for economic status, of lesser magnitude in comparison with our findings, for overweight and obesity [12]. India and Bangladesh similarly shows an increase in obesity prevalence with increase in education and standard of living index [9,11]. Nepal and Maldives reveals higher prevalence of overweight and obesity in lower educated groups while showing a clear social gradient for diabetes, hypertension and metabolic syndrome [8,10]. Epidemiological transitions were observed in Brazil where higher obesity prevalence observed among richest quintile was revered to the poorest quintile, in Sri Lanka such a change has not been observed for the past three decades despite its move from a LIC to a LMIC [30,32].

The higher prevalence of obesity in Muslim communities may indicate social and cultural practices that influence lifestyle and diet. This was not demonstrated in the two large surveys on obesity conducted in 2005/6 [7,25,]. However this is consistent with the high prevalence of diabetes and metabolic syndrome observed among Muslims in Sri Lanka [15,33]. Similarly in HICs high prevalence rates for obesity and diabetes was demonstrated among Asian migrants from Muslim countries [34,35]. Ethnicity and culture have not been explored as a risk factor in other South Asian countries.

Social gradient of obesity may contribute to the social gradient of NCDs. This is already observed with regard to diabetes mellitus including in Sri Lanka [15]. Those of higher socio economic status may access the limited healthcare facilities more than the lower categories and thus any regressive disparity in resource allocations may be exaggerated. This would in turn give rise to more complications and poor control of NCDs among the lower socioeconomic categories. This may explain the observed linear social gradient for mean BMI and mean WC among those with diabetes mellitus, possibly due to a more active response to illness by the higher socio economic group.

If economic growth continues, with the cessation of war and increased overseas investment, and the middle class expands, a larger proportion of the population may then be exposed to these risk factors with consequent increase in obesity and NCD rates [32]. NCD prevention in Sri Lanka should target all socio economic categories with higher emphasis on the wealthy, females and Muslim communities.

## Conclusion

Obesity in Kalutara District is higher among females, urban dwellers, high socio economic groups and in the Muslim community. A non linear social gradient of overweight, obesity and abdominal obesity is observed for education level, social status index levels, sector of residence and UBNI groups while income categories display a linear social gradient.

### Supplementary information

Supplementary information is available at http://www.nature.com/ijo/index.html.

## Additional files

Additional file 1:
**Reliability assessment of anthropometric measurements.**
Additional file 2:
**Inter–observer reliability of anthropometric measurements.**

## Abbreviations

BMI: Body Mass Index
HIC: High Income Country
LIC: Low Income Country
LMIC: Lower Middle Income Country
NCD: Non Communicable Diseases
PHNS: Public Health Nursing Sister
UBNI: Unsatisfactory Basic Needs Index
WC: Waist circumference
