# Supplementary material for: A cross sectional survey on social, cultural and economic determinants of obesity in a low middle income setting
Source: Int J Equity Health. 2015 Jan 17;14:6. doi: 10.1186/s12939-015-0140-8 (PMC4300585; doi:10.1186/s12939-015-0140-8)
Supplement: Additional file 1: — Reliability assessment of anthropometric measurements. [file 12939_2015_140_MOESM1_ESM.docx]

**Additional file 1:** Reliability assessment of anthropometric measurements

|  |  |  | **PHNS** |  |  | **Mean** | **Variance** |
| --- | --- | --- | --- | --- | --- | --- | --- |
|  | **1** | **2** | **3** | **4** | **5** |  |  |
| **Weight**^1^ |  |  |  |  |  |  |  |
| I1 | 48.5 | 71.75 | 80.9 | 64.3 | 56.61 | 64.41 | 159.94 |
| I2 | 56.6 | 48.45 | 71.7 | 80.9 | 64.36 | 64.4 | 160.2 |
| I3 | 64.5 | 56.65 | 48.5 | 71.6 | 80.96 | 64.44 | 159.74 |
| I4 | 81 | 64.3 | 56.5 | 48.5 | 71.73 | 64.41 | 161.13 |
| I5 | 71.9 | 81 | 64.4 | 56.6 | 48.5 | 64.48 | 161.36 |
| Mean | 64.5 | 64.43 | 64.4 | 64.38 | 64.43 |  |  |
| Variance | 161.36 | 161.01 | 160.19 | 159.44 | 160.36 |  |  |
| **Height**^1^ |  |  |  |  |  |  |  |
| I1 | 149.9 | 173.1 | 163.5 | 166.2 | 149.8 | 160.5 | 106.78 |
| I2 | 148.8 | 149.9 | 173 | 162.8 | 166.7 | 160.24 | 112.22 |
| I3 | 166 | 148.6 | 150.1 | 172.5 | 162.9 | 160.02 | 107.16 |
| I4 | 162.7 | 165.8 | 149 | 150.1 | 172.3 | 159.98 | 102.81 |
| I5 | 172.7 | 162.9 | 166 | 150 | 150 | 160.32 | 101.30 |
| Mean | 160.02 | 160.06 | 160.32 | 160.32 | 160.34 |  |  |
| Variance | 108.01 | 111.40 | 108.94 | 100.01 | 102.01 |  |  |
| **WC**^1^ |  |  |  |  |  |  |  |
| I1 | 70 | 93 | 100.5 | 84 | 87 | 86.9 | 129.05 |
| I2 | 86 | 70 | 94 | 100 | 85 | 87 | 128 |
| I3 | 83 | 88 | 73 | 92 | 99.5 | 87.1 | 98.55 |
| I4 | 96 | 81 | 89 | 70 | 95 | 86.2 | 117.7 |
| I5 | 92 | 96 | 84 | 87 | 70 | 85.8 | 99.2 |
| Mean | 85.4 | 85.6 | 88.1 | 86.6 | 87.3 |  |  |
| Variance | 99.8 | 108.3 | 108.55 | 122.8 | 128.2 |  |  |

**^1^**Inter observer variability: p=0.99, Inter instrument variability: p= 0.99, Inter subject variability: p= <0.0001
