# Supplementary material for: A cross sectional survey on social, cultural and economic determinants of obesity in a low middle income setting
Source: Int J Equity Health. 2015 Jan 17;14:6. doi: 10.1186/s12939-015-0140-8 (PMC4300585; doi:10.1186/s12939-015-0140-8)
Supplement: Additional file 2: — Inter–observer reliability of anthropometric measurements. [file 12939_2015_140_MOESM2_ESM.docx]

**Additional file 2:** Inter–observer reliability of anthropometric measurements

| **Measurement** | **Level of agreement between PI and PHNS** | | | | |
| --- | --- | --- | --- | --- | --- |
|  | **PHNS 1** | **PHNS 2** | **PHNS 3** | **PHNS 4** | **PHNS 5** |
| **Weight** | 0.97 | 0.97 | 0.98 | 0.99 | 0.98 |
| **Height** | 0.83 | 0. 83 | 0.91 | 0.94 | 0.91 |
| **Waist circumference** | 0.79 | 0.78 | 0.85 | 0.9 | 0.88 |
